# Au Modified F-TiO_2_ for Efficient Photocatalytic Synthesis of Hydrogen Peroxide

**DOI:** 10.3390/molecules26133844

**Published:** 2021-06-24

**Authors:** Lijuan Li, Bingdong Li, Liwei Feng, Xiaoqiu Zhang, Yuqian Zhang, Qiannan Zhao, Guifu Zuo, Xianguang Meng

**Affiliations:** 1 Hebei Provincial Laboratory of Inorganic Nonmetallic Materials, College of Materials Science and Engineering, North China University of Science and Technology, Tangshan 063210, China; Lilj5527@163.com (L.L.); libingdong1226@163.com (B.L.); 13473519550@163.com (L.F.); 18332737639@163.com (Y.Z.); 18332725160@163.com (Q.Z.); zuoguifu@163.com (G.Z.); 2 Chemistry Group, No.2 Experimental Middle School of Dehui, Dehui 130300, China; zhangxiaoqiu-913@163.com

**Keywords:** photocatalysis, hydrogen peroxide, fluorination, TiO_2_

## Abstract

In this work, Au-modified F-TiO_2_ is developed as a simple and efficient photocatalyst for H_2_O_2_ production under ultraviolet light. The Au/F-TiO_2_ photocatalyst avoids the necessity of adding fluoride into the reaction medium for enhancing H_2_O_2_ synthesis, as in a pure TiO_2_ reaction system. The F^−^ modification inhibits the H_2_O_2_ decomposition through the formation of the ≡Ti–F complex. Au is an active cocatalyst for photocatalytic H_2_O_2_ production. We compared the activity of TiO_2_ with F^−^ modification and without F^−^ modification in the presence of Au, and found that the H_2_O_2_ production rate over Au/F-TiO_2_ reaches four times that of Au/TiO_2_. In situ electron spin resonance studies have shown that H_2_O_2_ is produced by stepwise single-electron oxygen reduction on the Au/F-TiO_2_ photocatalyst.

## 1. Introduction

Hydrogen peroxide (H_2_O_2_) is widely used as a clean oxidant in environmental purification and organic synthesis [1,2]. It is widely used in pulp bleaching, wastewater treatment, and disinfection of industrial and household wastes with only water as the by-product [3]. At present, most H_2_O_2_ in industry is prepared by the anthraquinone method with H_2_ and O_2_ [4]. This method requires a lot of energy and organic solvents with complicated reaction steps and high risk of explosion. Therefore, finding a simple and direct method for H_2_O_2_ synthesis has become the focus of research. H_2_O_2_ can be effectively produced through photo-electrocatalysis [5] and photocatalysis. In recent years, the photocatalytic synthesis of H_2_O_2_ with oxygen and sunlight as the input energy has attracted great attention. At present, many semiconductor materials with UV and visible light activities, such as ZnO [6,7], C_3_N_4_ [8,9,10], BiVO_4_ [11], and TiO_2_ [12,13,14,15,16,17,18] have demonstrated the potential for direct synthesis of H_2_O_2_. Especially when these semiconductors are loaded with appropriate cocatalysts, the photocatalytic activity of the catalysts could be greatly improved. Au has been proved to be a very effective cocatalyst for promoting H_2_O_2_ production.

As a classic photocatalyst, TiO_2_ is one of the most frequent and promising semiconductors because of its low cost and high stability. Under UV irradiation, H_2_O_2_ can be directly produced in aqueous solution in the presence of O_2_ without hydrogen over TiO_2_. An important feature of photocatalytic H_2_O_2_ synthesis is that the formation of H_2_O_2_ from the oxygen reduction reaction (ORR) is accompanied by the decomposition process. Zhao et al. [19] reported that adsorption of H_2_O_2_ on TiO_2_ will readily form surface peroxide complexes in the form of ≡Ti–OOH, which can be easily photodegraded with a zero-order kinetic process, even with the irradiation of visible light, thus leading to the decrease in H_2_O_2_ production. Maurino et al. [20] also reported that the production of H_2_O_2_ increased remarkably after adding fluoride into the reaction suspension of TiO_2_. These studies showed the competition of the F^−^ with superoxide/peroxide species for the surface sites of TiO_2_. The ≡Ti–F formation decreases the amount of ≡Ti–OOH and thus, inhibits H_2_O_2_ degradation. This method is interesting but it will cause fluoride pollution to the reaction medium and the difficulty of H_2_O_2_ purification. In order to solve these problems, we developed F^−^-modified TiO_2_ by a hydrothermal method instead of adding NaF in the photocatalytic reaction medium and used Au as the cocatalyst of F-TiO_2_. The anchored F^−^ on the TiO_2_ surface will compete with the formation of peroxide species to suppress the decomposition of H_2_O_2_ and increase the H_2_O_2_ production rate. F-TiO_2_ avoided adding fluoride into the reaction medium as used in a pure TiO_2_ reaction system and thus, simplified the reaction procedure. In situ ESR reveals that the H_2_O_2_ is efficiently formed through a stepwise single-electron ORR process on the Au/F-TiO_2_ photocatalyst.

## 2. Materials and Methods

### 2.1. Experimental Materials Preparation

To produce the F-TiO_2_ photocatalyst, 1 g commercial anatase TiO_2_ and 0.42 g NaF (n_F_:n_Ti_ = 0.5:1) were mixed with 25 mL absolute ethanol and 15 mL water for hydrothermal treatment. The powder mixtures were maintained at 180 °C for 4 h in a homogeneous reactor. Then, the mixtures were transferred into the deionized water for centrifugation, washing and drying. By changing the amount of NaF and TiO_2_ with different molar ratios of F/Ti, we prepared a series of F-TiO_2_ photocatalysts. The photocatalysts loaded with 0.1 wt% Au were obtained by the deposition–precipitation method reported previously [21].

### 2.2. Material Characterization

UV–Vis spectra were recorded with a Spectrum Lambda 750 S (Perkin-Elmer, Waltham, MA, USA). High-resolution transmission electron microscopy (TEM) characterization was performed with an 8000EX microscope (JEOL, Tokyo, Japan) operating at 200 kV. The S-4800 scanning electron microscope (SEM) from Hitachi Instruments was used to observe the morphology of the photocatalyst.

### 2.3. Photocatalytic Activity Test

A reaction kettle (200 mL) was used as a photocatalytic reactor; 0.2 g Au/F-TiO_2_ was added into the reaction solution of alcohol (4 wt%) and deionized water. F^−^ was directly modified on the surface of the TiO_2_ by the hydrothermal method without adding F^−^ into the reaction solution. The suspension was treated by ultrasonication for 2–3 min; then, oxygen was bubbled for 30 min before turning on the light. A 300 W Xe arc lamp (PLS-SXE300, Perfectlight Technology Co., Ltd., Beijing, China) was used as a light source. The reaction was carried out under magnetic stirring water cooling. The concentrations of H_2_O_2_ generated were determined by using the DMP (2, 9-dimethyl-1, 10-phenanthroline) method [22].

### 2.4. Quantification of H_2_O_2_ (DMP Method)

One milliliter of DMP (0.1 g/L), 1 mL of copper (II) sulfate (0.1 M), 1 mL of phosphate buffer (Ph 7.0) solution, and 1 mL of reaction solution were added to a 10 mL volumetric flask and mixed; then, deionized water was added to the volumetric flask to the tick mark. After mixing, the absorbance of the sample at 454 nm was measured. The blank solution was prepared in the same manner but without H_2_O_2_.

The concentrations of H_2_O_2_ were calculated by the following formula:

A_454_ = ζ [H_2_O_2_] × 1/10

where A_454_ is the difference of the absorbance between the sample and blank solutions at 454 nm, ζ is the slope of the calibration curve, and [H_2_O_2_] is the H_2_O_2_ concentration (µM).

### 2.5. In Situ ESR Test

Using 5,5-dimethyl-1-pyrroline N-oxide (DMPO) as spin trapping reagent, the reduction pathways of O_2_ on different catalysts were determined by in situ electron spin resonance (ESR) analysis. An ESP 300E spectrometer (Bruker, Switzerland) was used to detect the ESR signals of radicals trapped by DMPO. Generally, the catalyst (1 mg) was put into a mixture containing 1 mL alcohol/water (4 wt%) and 0.125 mmol DMPO. After passing the O_2_ for 3 min, the sample was irradiated under UV light for 5 min before testing.

## 3. Results and Discussion

**Figure 1:**
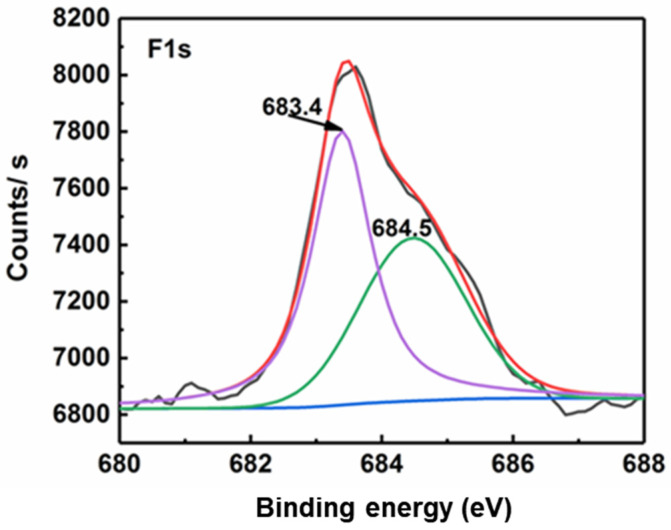
High-resolution XPS spectrum of F1s.

Au/TiO_2_ and TiO_2_ have similar XRD test spectra (Figure S1). The diffraction peak of Au was not observed. It is presumed that the content of Au is too low and it is highly dispersed in the catalyst, which makes it impossible to form obvious characteristic diffraction peaks. In order to explore the existence and state of F^−^ in the catalyst, the XPS of Au/TiO_2_ and Au/F-TiO_2_ was tested (Figure S2). Compared with the full spectrum of Au/TiO_2_, the full spectrum of Au/F-TiO_2_ has a peak corresponding to F1s between 600 eV and 700 eV, which can preliminarily prove that F^−^ has been successfully introduced into the catalyst. From the peak fitting results of the high-resolution XPS spectrum of F1s (Figure 1), it is found that the F1s is mainly composed of two peaks. The low binding energy peak at 683.4 eV is the signal peak of the formation of complex ≡Ti-F due to the chemical adsorption of F^−^ on the surface of TiO_2_. The small peak with high binding energy near 684.5 eV is attributed to the signal peak of the doped F atom in TiO_2_; that is, the F atom substituting for the oxygen site in TiO_2_ lattice.

**Figure 2:**
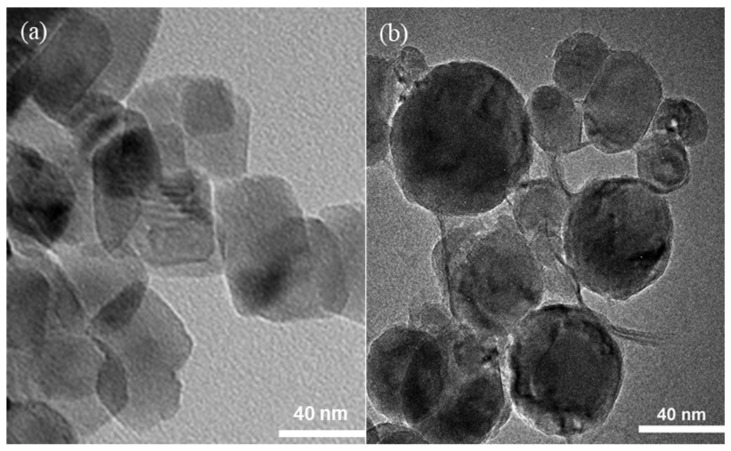
TEM images of (**a**) TiO_2_ and (**b**) F-TiO_2_.

From the TEM image of TiO_2_ and F-TiO_2_ (Figure 2), it is found that the morphology of F-TiO_2_ prepared by the hydrothermal method has a very obvious change compared with that of TiO_2_. TiO_2_ has an irregular shape, while F-TiO_2_ is almost spherical. This is because F^−^ has an etching effect on TiO_2_ during hydrothermal treatment [23]. The F^−^ has a strong complexation ability with Ti on the surface of TiO_2_, which corrodes the edges and corners of TiO_2_ particles and changes the irregular TiO_2_ into a spherical shape [24].

**Figure 3:**
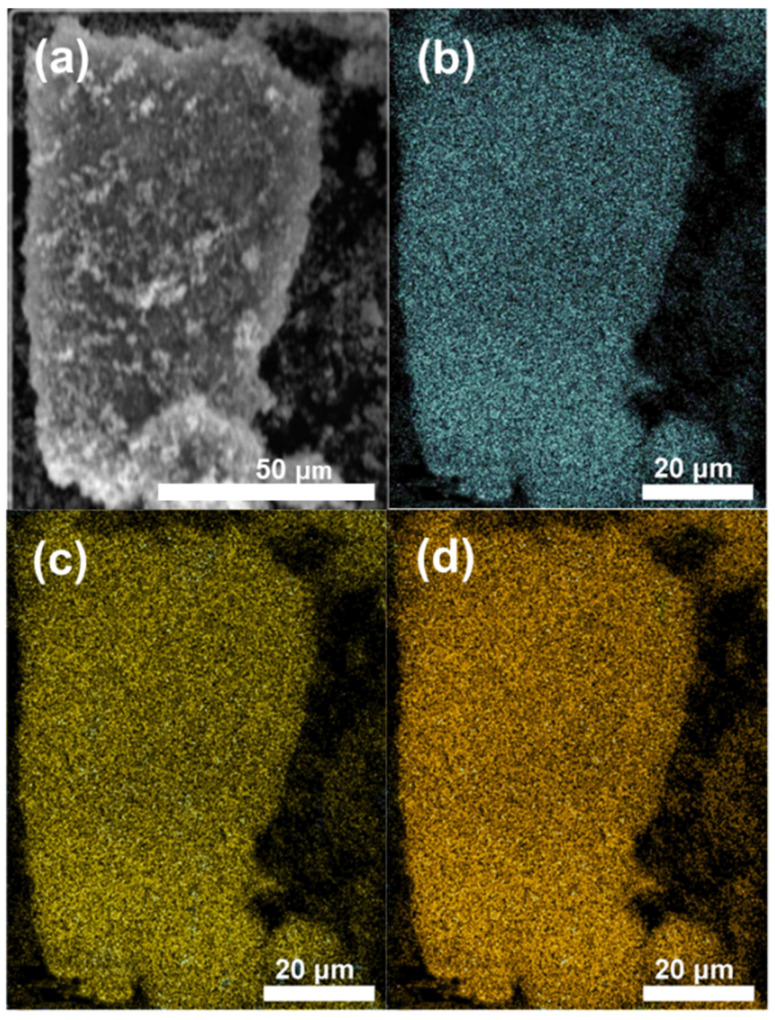
(**a**) SEM and mapping images of (**b**) Ti, (**c**) F and (**d**) Au elements on Au/F-TiO_2_ (n_F_:n_Ti_ = 2.5).

The SEM and energy dispersive spectroscopy (EDS) of 0.1% Au/F-TiO_2_ (Figure S3) show that there are F and Au elements on the surface of the catalyst. This also proved that the F^−^ modification and Au loading on TiO_2_ were successfully realized in the sample preparation. The element mapping of Au/F-TiO_2_ (n_F_: n_Ti_ = 2.5) in Figure 3 shows that both F and Au are evenly distributed on the surface of TiO_2_, which is consistent with the element types shown in the EDS result (Figure S3).

**Figure 4:**
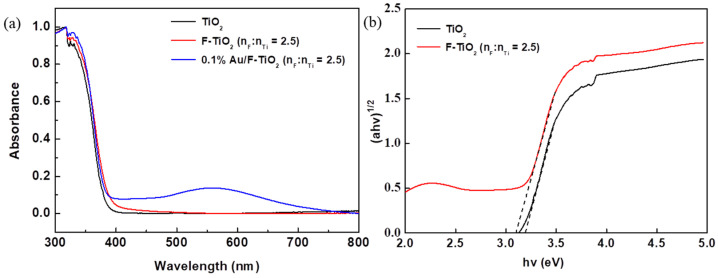
(**a**) UV–Vis absorbance from Kubelka–Munk function of diffuse–reflectance spectra of TiO_2_, F-TiO_2_ and 0.1% Au/F-TiO_2_; (**b**) Tauc plot from (**a**).

Figure 4a shows the UV–Vis spectrum of F-TiO_2_, 0.1% Au/F-TiO_2_ and pure TiO_2_. Besides the characteristic absorption bands of TiO_2_ at lower than 370 nm, the absorption bands caused by the loading of Au nanoparticles are located between 500 nm and 650 nm, which is a typical Au surface plasma band [25]. According to the calculation, the band gap of TiO_2_ is about 3.2 eV and F-TiO_2_ is about 3.1 eV (Figure 4b).

**Figure 5:**
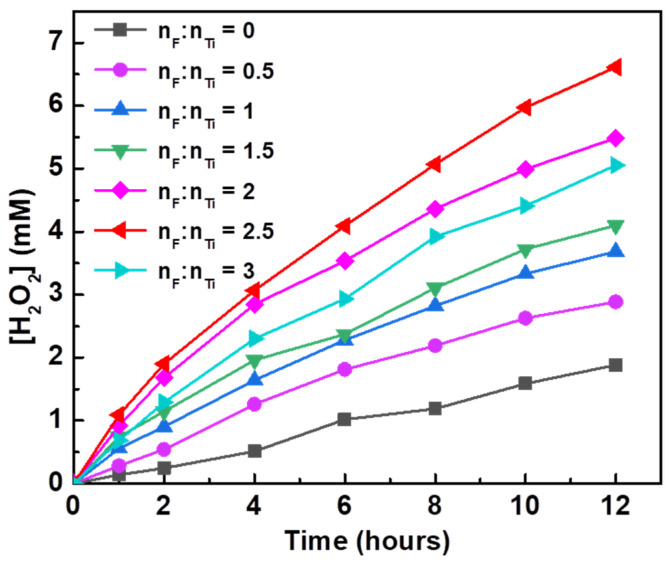
Photocatalytic H_2_O_2_ production over 0.1% Au/F-TiO_2_ prepared with different F/Ti ratios.

The photocatalytic activity of H_2_O_2_ synthesis on Au/F-TiO_2_ hybrids was tested under UV light and the concentration of H_2_O_2_ was quantified by spectrophotometry with copper ions and 2,9-dimethyl-1,10-phenanthroline (DMP). The standard curve showed that there was a good linear relationship between the absorbance and concentration of H_2_O_2_; the R squared value was 0.9996 (Figure S3). Figure 5 shows the photocatalytic synthesis of H_2_O_2_ over Au-loaded F-TiO_2_ catalysts. Compared with the unmodified catalyst, the photocatalytic activity increased with the increase in F content, and the photocatalytic activity reached its highest when the F/Ti molar ratio increased to 2.5. The F^−^ on the surface of TiO_2_ will compete with superoxide/peroxide species for the surface sites of TiO_2_ and inhibit the adsorption of peroxy radicals, thus suppressing the decomposition of H_2_O_2_. With the continuous increase in F content, when the F/Ti molar ratio is 3, the activity of the catalyst decreases. Excessive F^−^ caused serious defects on the surface of TiO_2_ and destroyed the crystallinity of TiO_2_, thus decreasing the photocatalytic activity of the catalyst.

**Figure 6:**
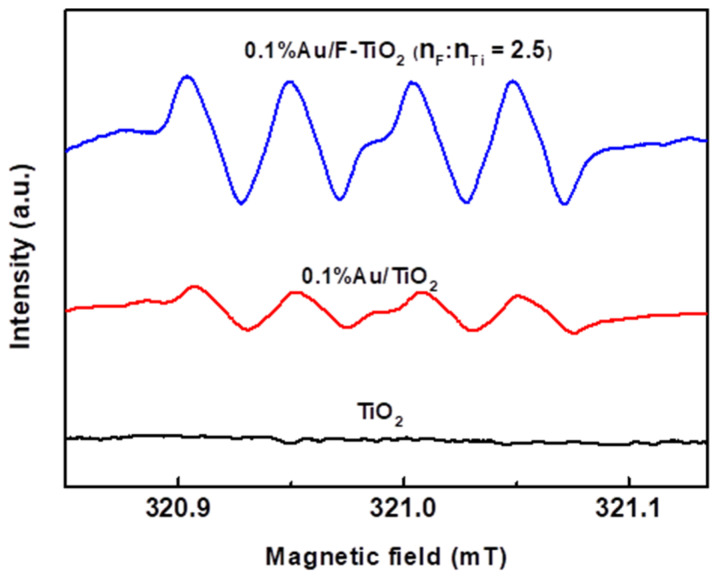
In situ ESR spectra of 0.1% Au/TiO_2_ prepared with different F/Ti ratios and pure TiO_2_.

In general, H_2_O_2_ from 2 e^−^ ORR by CB electrons can be produced through stepwise coupled electrons and proton transfers (Equations (1)–(3)) [26]. In order to further study the mechanism of photocatalytic ORR for H_2_O_2_ synthesis over Au/F-TiO_2_, DMPO was used as a trapping agent of free radical in situ ESR tests for different samples. In situ ESR spectra of Au/F-TiO_2_, Au/TiO_2_ and pure TiO_2_ under UV irradiation are shown in Figure 6. The results clearly show the signal of •OOH formed via equation (2) over various TiO_2_ photocatalysts [27]. The DMPO-•OOH radical signal could be detected in both Au-loaded samples except pure TiO_2_. The superoxide radical is formed by the first combination of O_2_ in the photocatalytic reaction medium with electrons and protons. The generated HO_2_• will continue to react with one electron and a proton and finally, generate H_2_O_2_. Therefore, the H_2_O_2_ is formed by a stepwise single-electron ORR over Au/F-TiO_2_. In addition, compared with TiO_2_, Au/TiO_2_ and Au/F-TiO_2_ produced a more obvious HO_2_• signal, which implied that Au and F^−^ promoted the formation of HO_2_•, and both of them promoted the photocatalytic synthesis of H_2_O_2_.

Semiconductor → e^−^ + h^+^ (1)

e^−^ + O_2_ + H^+^ → •OOH (2)

•OOH + H^+^ + e^−^ → H_2_O_2_ (3)

## 4. Conclusions

In this work, we have designed Au/F-TiO_2_ as an efficient photocatalyst for the production of H_2_O_2_ in aqueous solution. The Au/F-TiO_2_ makes it possible to obtain a high H_2_O_2_ yield in fluoride-free reaction medium. The H_2_O_2_ production rate reached four times that of Au/TiO_2_. The in situ ESR test showed that the synthesis mechanism of H_2_O_2_ was not changed by F^−^ modification. The H_2_O_2_ was synthesized over Au/F-TiO_2_ through a stepwise single-electron ORR.

## Supplementary Materials

The following are available online. Figure S1: XRD spectra of Au/TiO_2_ and TiO_2_, Figure S2: XPS spectra of (a) Au/TiO_2_, (b) Au/F-TiO_2_, Figure S3: (a) SEM image of 0.1%Au/F-TiO_2_, (b) EDS spectrum of 0.1%Au/F-TiO_2_, Figure S4: Standard curve: linear relationship between absorbance at 454 nm and H_2_O_2_ concentration.

## Sample Availability

Not available.
