# Supplementary material for: Au Modified F-TiO2 for Efficient Photocatalytic Synthesis of Hydrogen Peroxide
Source: Molecules. 2021 Jun 24;26(13):3844. doi: 10.3390/molecules26133844 (PMC8270298; doi:10.3390/molecules26133844)
Supplement: Supplementary file 1 [file molecules-26-03844-s001.zip › molecules-1240480-SI.pdf]

Surporting information

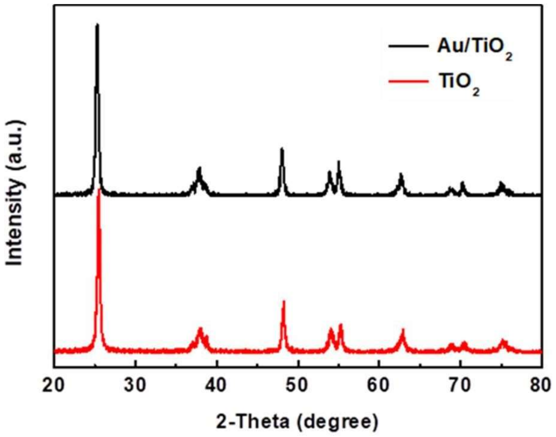

Figure S1. XRD spectra of Au/TiO_2 and TiO_2.

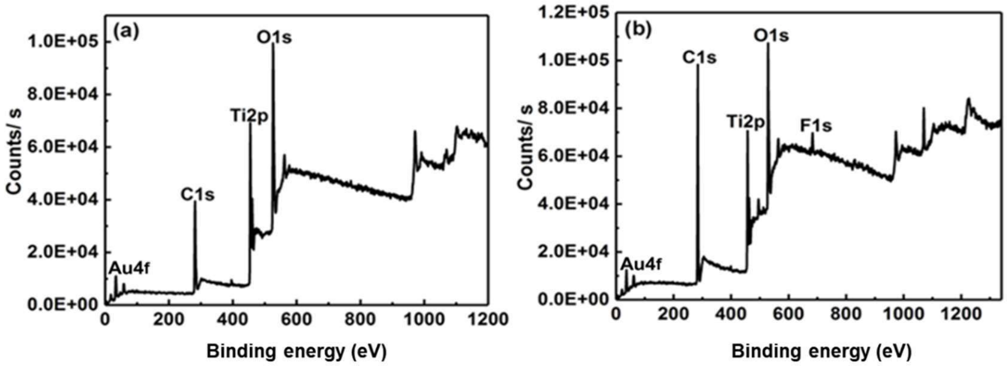

Figure S2. XPS spectra of (a) Au/TiO_2, (b) $Au/F\text{-}TiO_2$.

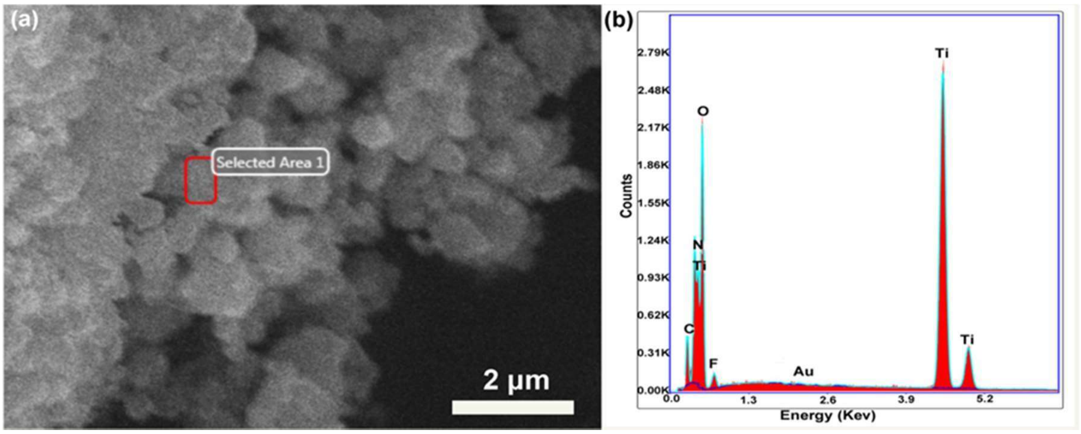

Figure S3. (a) SEM image of 0.1%$Au/F\text{-}TiO_2$, (b) EDS spectrum of 0.1%$Au/F\text{-}TiO_2$.

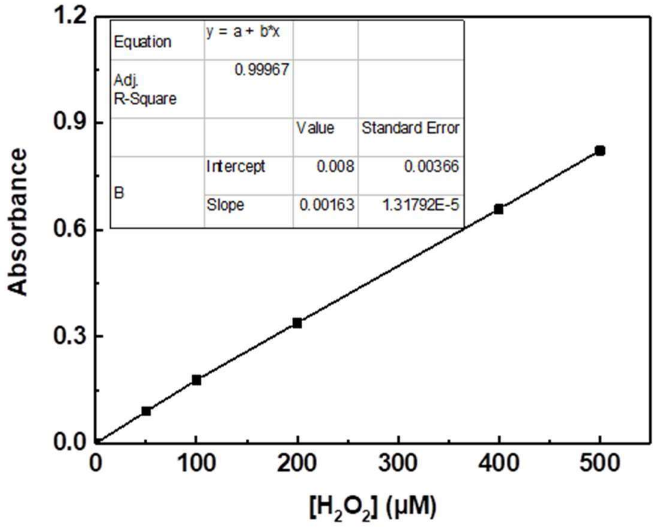

Figure S4. Standard curve: linear relationship between absorbance at 454 nm and H_2O_2 concentration.
